# Synthesis and crystal structure of cerium(IV) bis­(phosphite)

**DOI:** 10.1107/S205698901701115X

**Published:** 2017-08-04

**Authors:** Stefano H. Byer, Eric M. Villa

**Affiliations:** a Department of Chemistry, Creighton University, 2500 California Plaza, Omaha, Nebraska 68178, USA

**Keywords:** crystal structure, cerium(IV), phosphite

## Abstract

The structure of cerium(IV) bis­(phosphite), Ce(HPO_3_)_2_, has been solved by single-crystal X-ray diffraction and has trigonal (*P*-3*m*1) symmetry. The cerium(IV) cation exhibits site symmetry -3*m*. and is octa­hedrally coordinated by O atoms of the phosphite ligands (point group symmetry 3*m*.). The highly symmetrical compound has a layered structure parallel to the *ab* plane, and is closely related to zirconium(IV) bis­(phosphite) solved *via* powder X-ray diffraction with trigonal (*P*-3 symmetry. Structural details of the two compounds are comparatively discussed.

## Abstract 2

The title substance of cerium(IV) bis­(phosphite), Ce(HPO_3_)_2_, crystallizes in a trigonal cell and is composed of CeO_6_ octa­hedra and phosphite tetra­hedra.

## Chemical context   

Phospho­nates are commonly employed within the petroleum industry as anti­oxidants. Inter­actions between these anti­oxidants and possible metal impurities could potentially have unintended consequences in the processing of petroleum products. We are currently studying these inter­actions by exploring the crystalline materials formed *via* solvothermal syntheses of lanthanides with phospho­rous acid.

Phospho­rous acid (H_3_PO_3_) is a powerful reducing agent and is exceedingly water soluble. The anion HPO_3_^2−^ has many different names in the literature, including (but not limited to) phosphite, phospho­nate, phospho­rus(III) oxoanion, oxophosphate(III) and hydridotrioxidophosphate(2-). According to IUPAC, when the hydrogen atom is directly bonded to the phospho­rus atom, it is to be named phospho­nate; whereas when the anion tautomerizes to the PO_2_(OH)^2−^ anion, it is named as phosphite. However, the latter ion is rarely identified in the solid state. While IUPAC prefers HPO_3_^2−^ to be named phospho­nate, this name is also used for organo­phospho­rus compounds with the general formula *R*-PO(OH)_2_ or *R*-PO(OR)_2_, where *R* = alkyl or aryl groups. To eliminate any confusion with organo­phospho­rus compounds and to be consistent with the recent literature, we will herein refer to the HPO_3_^2−^ anion as phosphite.

The phosphite anion has many structural similarities to both phosphates and organo­phospho­nates. In the phosphite anion, a hydrogen atom has replaced one of the oxygen atoms, which would be found in phosphate; also, the phosphite anion contains no P—C bonds that are found in phospho­nates. Phosphite itself can be used as a precursor for making a vast array of phospho­nates. In phosphite, the central phospho­rus atom is P^III^ instead of the more air-stable P^V^, which provides the opportunity for redox chemistry. This ability to act as a reducing agent has led to several mixed-valent uranium compounds (Villa *et al.*, 2012 ▸; Villa, Marr *et al.*, 2013 ▸; Villa, Alekseev *et al.*, 2013 ▸). Moving towards the lanthanides, lanthanide phosphite compounds have been synthesized in one of two main ways: hydro­thermally (Cross *et al.*, 2012 ▸; Ewald *et al.*, 2003 ▸, 2005 ▸; Foulon *et al.*, 1993*a* ▸,*b* ▸,1995 ▸; Loukili *et al.*, 1988 ▸, 1991 ▸; Tijani *et al.*, 1988 ▸; Xiong *et al.*, 2006 ▸, 2009 ▸; Zhang *et al.*, 1992 ▸) and phosphite flux reactions (Zakharova *et al.*, 2003 ▸). We have expanded this chemistry by exploring solvothermal syntheses of lanthanide phosphites. Herein we will discuss the crystal structure of the title compound, a new cerium(IV) bis­(phosphite).

## Structural commentary   

**Figure 1:**
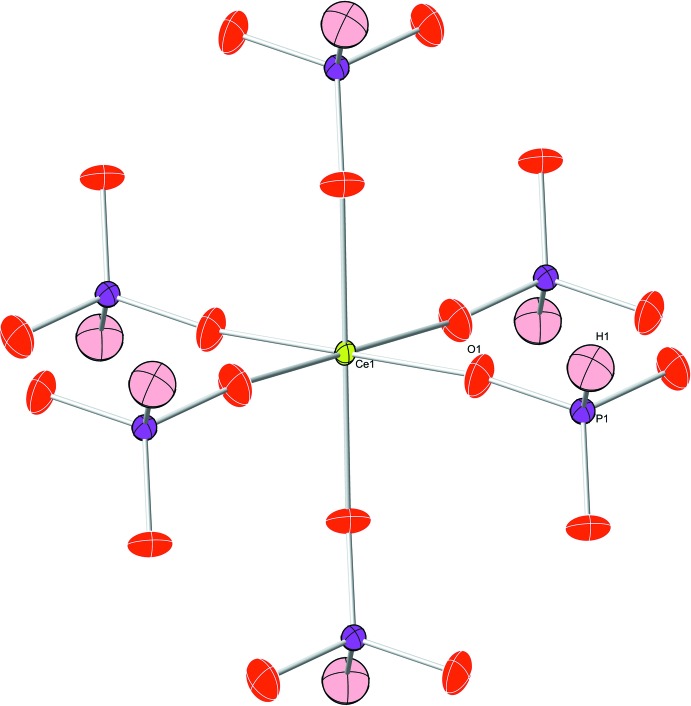
The coordination sphere of the cerium(IV) atom with atoms of the asymmetric unit labelled. Displacement ellipsoids are drawn at the 50% probability level. Bond lengths and angles can be found in Table 1 ▸.

**Figure 2:**
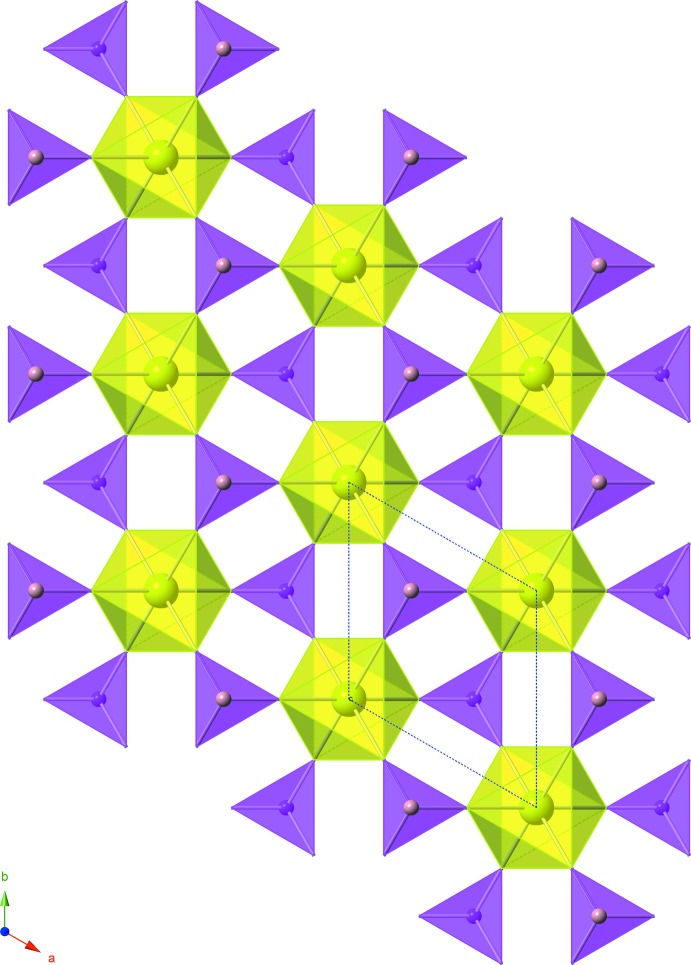
The sheet-like arrangement of the polyhedra parallel to the *ab plane* in the crystal structure of Ce(HPO_3_)_2_. The unit cell is shown with blue dashed lines. This polyhedral representation contains the same color scheme as Fig. 1 ▸.

**Figure 3:**
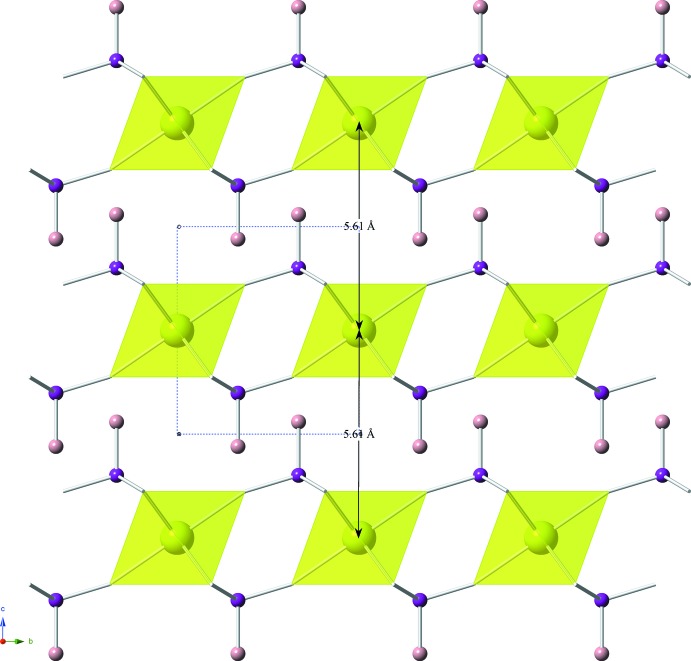
The stacking of the cerium(IV) phosphite sheets are shown in a projection along the *a* axis. Again, the unit cell is shown with blue dashed lines and the color scheme is the same as the above images.

The title compound Ce(HPO_3_)_2_ crystallizes in the space group *P* 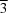 *m*1. The smallest repeating unit contains one cerium(IV), one oxygen, one phospho­rus(III) and one hydrogen atom. This simple structure contains slightly distorted octa­hedrally coordinated cerium(IV) cations (site symmetry 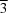 *m*.; Fig. 1 ▸), which are linked together by corner-sharing phosphite ligands. These phosphite ligands have a slightly distorted tetra­hedral configuration (point group symmetry 3*m*.). Each phospho­rus(III) atom in the phosphite ligand is bonded to three oxygen atoms, comprising the bottom of the tetra­hedron, and one hydrogen atom. The sheets of Ce(HPO_3_)_2_, which are located in the *ab* plane, contain alternating up–down phosphite ligands around the cerium(IV) metal cation, as indicated by the different directions of the hydrogen atoms (Figs. 2 ▸, 3 ▸). These sheets are layered down the *c* axis, where each cerium(IV) atom resides directly below the cerium above it at a distance of 5.6099 (3) Å, which corresponds to the length of the *c* axis; between the layers, the Ce—Ce—Ce angle is 180.0° (Fig. 3 ▸).

This structure is closely related to a known zirconium(IV) bis­(phosphite), Zr(HPO_3_)_2_, which was isolated *via* a very different synthesis route involving refluxing in concentrated phospho­rous acid and using HF to precipitate the desired compound (Millini *et al.*, 1993 ▸). The structure was elucidated from powder X-ray diffraction data and has many similarities to the title structure shown above (Table 1 ▸). The main difference between the two is the metal–oxygen bond length, where the Ce—O bond length is unsurprisingly slightly longer. The zirconium coordination appears to be a bit closer to being octa­hedral than the cerium, but they are nearly the same after comparing the error of refinement.

## Synthesis and crystallization   

Cerium(IV) bis­(phosphite) was synthesized solvothermally in aceto­nitrile (CH_3_CN). A stock solution of 1.00 M H_3_PO_3_ was prepared in aceto­nitrile. 0.0572 grams of ceric ammonium nitrate, (NH_4_)_2_Ce(NO_3_)_6_, was placed into a PTFE liner along with 2 ml of the 1.00 *M* phospho­rous acid solution, yielding a solution 0.0522 M ceric ammonium nitrate. This gives an approximate molar ratio of cerium(IV) to phosphite of 1:20. After the cerium was completely dissolved, the PTFE liner was capped and sealed inside of a stainless steel autoclave. This was then placed into a programmable box furnace and heated to 363 K over a period of 30 minutes, held at 363 K for four h and then cooled for 975 minutes down to 298 K (or a rate of 4 K per hour).

The resulting mixture was washed with cold water and then placed into a plastic petri dish. The excess water was removed and the crystals were dispersed with aceto­nitrile. The large, hexa­gonal prisms of the title compound were light yellow in color. Many suitable crystals were present. A large crystal was isolated in immersion oil and broken perpendicular to the hexa­gonal face to yield a clean crystal for single-crystal X-ray diffraction.

## Refinement   

Crystal data, data collection and structure refinement details are summarized in Table 2 ▸. The hydrogen-atom position was placed as a riding atom on the phospho­rus position. The maximum electron density peaks are 0.330 e^−^ Å^−3^ (located between P1 and O1 at 0.686 and 0.838 Å, respectively), 0.320 e^−^ Å^−3^ (located adjacent to O1 at 0.608 Å) and 0.250 e Å^−3^ (located adjacent to Ce1 at 0.692 Å), which lead to nothing reasonable. All other maximum density peaks are under 0.2 e^−^ Å^−3^. The minimum electron density is a very minimal at −0.609 e^−^ Å^−3^. Two electron density holes of about the same magnitude reside around the phospho­rus atom.

## Supplementary Material

Crystal structure: contains datablock(s) I. DOI: 10.1107/S205698901701115X/wm5407sup1.cif

CCDC reference: 1565300

Additional supporting information:  crystallographic information; 3D view; checkCIF report

## Figures and Tables

**Table 1:** Comparison of bond lengths and angles (Å, °) in Ce(HPO_3_)_2_ (*P* 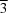 *m*1) and Zr(HPO_3_)_2_ (*P* 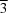)

Bond lengths	Ce(HPO_3_)_2_	Zr(HPO_3_)_2_	bond angles	Ce(HPO_3_)_2_	Zr(HPO_3_)_2_
Metal—O	2.2193 (19)	2.05 (2)	O—metal—O	88.85 (8), 91.15 (8)	89.2 (4), 90.8 (4)
P—O	1.5168 (19)	1.52 (1)	metal—O—P	162.28 (14)	162.1 (3)
P—H	1.44 (6)	1.43 (fixed)	O—P—O	112.07 (8)	111.2 (5)
			O—P—H	106.73 (9)	107.6 (6)

Zr(HPO_3_)_2_ data from Millini *et al.* (1993 ▸).

**Table 2:** Experimental details

Crystal data	
Chemical formula	Ce(HPO_3_)_2_
*M*_r_	300.08
Crystal system, space group	Trigonal, *P* 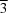 *m*1
Temperature (K)	293
*a*, *c* (Å)	5.6859 (3), 5.6099 (3)
*V* (Å^3^)	157.07 (2)
*Z*	1
Radiation type	Mo *K*α
μ (mm^−1^)	7.71
Crystal size (mm)	0.21 × 0.09 × 0.08

Data collection	
Diffractometer	Rigaku SCX-Mini
Absorption correction	Multi-scan (*CrysAlis PRO*; Rigaku OD, 2015 ▸)
*T*_min_, *T*_max_	0.799, 1.000
No. of measured, independent and observed [*I* > 2σ(*I*)] reflections	1852, 197, 197
*R*_int_	0.018
(sin θ/λ)_max_ (Å^−1^)	0.713

Refinement	
*R*[*F* ^2^ > 2σ(*F* ^2^)], *wR*(*F* ^2^), *S*	0.010, 0.029, 1.26
No. of reflections	197
No. of parameters	13
H-atom treatment	Only H-atom coordinates refined
Δρ_max_, Δρ_min_ (e Å^−3^)	0.51, −0.35

Computer programs: *CrysAlis PRO* (Rigaku OD, 2015 ▸), *SHELXT* (Sheldrick, 2015*a* ▸), *SHELXL2014* (Sheldrick, 2015*b* ▸), *OLEX2* (Dolomanov *et al.*, 2009 ▸) and *CrystalMaker* (Palmer, 2014 ▸),.

## supplementary crystallographic information

### Crystal data

**Table:**

Ce(HPO_3_)_2_	*D*_x_ = 3.172 Mg m^−^^3^
*M**_r_* = 300.08	Mo *K*α radiation, λ = 0.71073 Å
Trigonal, *P*3*m*1	Cell parameters from 1912 reflections
*a* = 5.6859 (3) Å	θ = 4.0–32.7°
*c* = 5.6099 (3) Å	µ = 7.71 mm^−^^1^
*V* = 157.07 (2) Å^3^	*T* = 293 K
*Z* = 1	Hexagonal, clear light yellow
*F*(000) = 138	0.21 × 0.09 × 0.08 mm

### Data collection

**Table:**

Rigaku SCX-Mini diffractometer	197 independent reflections
Radiation source: fine-focus sealed X-ray tube, Enhance (Mo) X-ray Source	197 reflections with *I* > 2σ(*I*)
Graphite monochromator	*R*_int_ = 0.018
ω scans	θ_max_ = 30.5°, θ_min_ = 5.5°
Absorption correction: multi-scan (*CrysAlis PRO*; Rigaku OD, 2015)	*h* = −8→8
*T*_min_ = 0.799, *T*_max_ = 1.000	*k* = −8→8
1852 measured reflections	*l* = −7→8

### Refinement

**Table:**

Refinement on *F*^2^	Primary atom site location: dual
Least-squares matrix: full	Hydrogen site location: difference Fourier map
*R*[*F*^2^ > 2σ(*F*^2^)] = 0.010	Only H-atom coordinates refined
*wR*(*F*^2^) = 0.029	*w* = 1/[σ^2^(*F*_o_^2^) + (0.0148*P*)^2^ + 0.0962*P*] where *P* = (*F*_o_^2^ + 2*F*_c_^2^)/3
*S* = 1.26	(Δ/σ)_max_ < 0.001
197 reflections	Δρ_max_ = 0.51 e Å^−^^3^
13 parameters	Δρ_min_ = −0.35 e Å^−^^3^
0 restraints	

### Special details

**Table:**

Geometry. All esds (except the esd in the dihedral angle between two l.s. planes) are estimated using the full covariance matrix. The cell esds are taken into account individually in the estimation of esds in distances, angles and torsion angles; correlations between esds in cell parameters are only used when they are defined by crystal symmetry. An approximate (isotropic) treatment of cell esds is used for estimating esds involving l.s. planes.
Refinement. The structure was refined using SHELXT (Sheldrick, 2015) Intrinsic Phasing and SHELXL (Sheldrick, 2015). Olex2 (Dolomanov *et al.*, 2009) was used as a graphical interface. Images of the above compound were made using CrystalMaker for Windows, version 9.2.8 (CrystalMaker, 2017). The refinement proceeded without any incidents and without any need for modelling disorder or twinning or restraints. The crystal was then mounted on MiTeGen Microloop with non-drying immersion oil. The crystal was then optically aligned on the Rigaku SCX-Mini diffractometer using a digital camera. Initial matrix images were collected to determine the unit cell, validity and proper exposure time. Three hemispheres (where φ= 0.0, 120.0 and 240.0) of data were collected with each consisting 180 images each with 1.00° widths and a 1.00° step. Refinement of the structure was based on F2 against all reflections. The R-factor R is based on F2>2σ(F2), but is not relevant to the choice of reflections for refinement; whereas the weighted R-factor wR and goodness of fit S are based on F2. Due to the presence of exclusively intense diffraction peaks, there is no observable difference between the R factors for all versus gt.

### Fractional atomic coordinates and isotropic or equivalent isotropic displacement parameters (Å^2^)

**Table:**

	*x*	*y*	*z*	*U*_iso_*/*U*_eq_	
Ce1	1.0000	1.0000	0.5000	0.01612 (11)	
P1	0.6667	0.3333	0.19839 (17)	0.01440 (18)	
O1	0.81417 (19)	0.6283 (4)	0.2762 (4)	0.0330 (4)	
H1	0.6667	0.3333	−0.059 (11)	0.040*	

### Atomic displacement parameters (Å^2^)

**Table:**

	*U*^11^	*U*^22^	*U*^33^	*U*^12^	*U*^13^	*U*^23^
Ce1	0.00810 (11)	0.00810 (11)	0.03215 (17)	0.00405 (6)	0.000	0.000
P1	0.0114 (3)	0.0114 (3)	0.0204 (4)	0.00569 (13)	0.000	0.000
O1	0.0330 (8)	0.0141 (8)	0.0456 (9)	0.0071 (4)	−0.0037 (4)	−0.0074 (7)

### Geometric parameters (Å, º)

**Table:**

Ce1—O1	2.2193 (19)	P1—H1	1.44 (6)
P1—O1	1.5168 (19)		
			
O1^i^—Ce1—O1^ii^	180.0	O1^ii^—Ce1—O1	91.15 (8)
O1^iii^—Ce1—O1^iv^	88.85 (8)	O1^i^—Ce1—O1^v^	91.15 (8)
O1^i^—Ce1—O1^iv^	88.85 (8)	O1^ii^—Ce1—O1^iv^	91.15 (8)
O1—Ce1—O1^iv^	91.15 (8)	O1—Ce1—O1^v^	88.85 (8)
O1—Ce1—O1^iii^	180.00 (9)	O1^vi^—P1—O1^vii^	112.07 (8)
O1^iii^—Ce1—O1^v^	91.15 (8)	O1^vi^—P1—O1	112.07 (8)
O1^i^—Ce1—O1^iii^	91.15 (8)	O1^vii^—P1—O1	112.07 (8)
O1^i^—Ce1—O1	88.85 (8)	O1—P1—H1	106.73 (9)
O1^ii^—Ce1—O1^iii^	88.85 (8)	O1^vi^—P1—H1	106.73 (9)
O1^ii^—Ce1—O1^v^	88.85 (8)	O1^vii^—P1—H1	106.73 (9)
O1^iv^—Ce1—O1^v^	180.0	P1—O1—Ce1	162.28 (14)

Symmetry codes: (i) *x*−*y*+1, *x*, −*z*+1; (ii) −*x*+*y*+1, −*x*+2, *z*; (iii) −*x*+2, −*y*+2, −*z*+1; (iv) −*y*+2, *x*−*y*+1, *z*; (v) *y*, −*x*+*y*+1, −*z*+1; (vi) −*x*+*y*+1, −*x*+1, *z*; (vii) −*y*+1, *x*−*y*, *z*.
